# Analysis of Cynandione A’s Anti-Ischemic Stroke Effects from Pathways and Protein-Protein Interactome

**DOI:** 10.1371/journal.pone.0124632

**Published:** 2015-05-08

**Authors:** Haiyang Fang, Rongcai Yue, Yang Ga, Yi Zhang, Lei Shan, Jing Zhao

**Affiliations:** 1 Department of Mathematics, Logistical Engineering University, Chongqing, China; 2 Department of Natural Medicinal Chemistry, Second Military Medical University, Shanghai, China; 3 School of Pharmacy, Shanghai Jiao Tong University, Shanghai, China; 4 Tibet Traditional Medical College, Lhasa, China; 5 The National Medical College, Chengdu University of TCM, Chengdu, China; Federico II University of Naples, ITALY

## Abstract

Ischemic stroke is the third leading cause of death in the world. Our previous study found that cynandione A (CYNA), the main component from the root of *Cynanchum bungei*, exhibits anti-ischemic stroke activity. In this work, we investigated the therapeutic mechanisms of CYNA to ischemic stroke at protein network level. First, PC12 cells and cerebellar granule neurons were prepared to validate the effects of CYNA against glutamate injury. Our experiments suggested that CYNA could dose-dependently mitigate glutamate-induced neurons neurotoxicity and inhibit glutamate-induced upregulation of KHSRP and HMGB1, further confirming the neuroprotective effects of CYNA *in vivo*. Then, on the pathway sub-networks, which present biological processes that can be impacted directly or in periphery nodes by drugs via their targets, we found that CYNA regulates 11 pathways associated with the biological process of thrombotic or embolic occlusion of a cerebral artery. Meanwhile, by defining a network-based anti-ischemic stroke effect score, we showed that CYNA has a significantly higher effect score than random counterparts, which suggests a synergistic effect of CYNA to ischemic stroke. This study may shed new lights on the study of network based pharmacology.

## Introduction

Stroke, also known as cerebrovascular insult (CVI) and cerebrovascular accident (CVA), is the brain malfunction resulted from insufficient blood supply to the brain [1]. This malfunction is brought about by either ischemia or hemorrhage. Ischemic stroke, featuring the abrupt interrupt of blood transportation to an portion of the brain, leads to the neurologic malfunction, which can cause permanent neurological damage or death and earns itself the title of the third killer of health worldwide in 2010 [2,3]. Ischemic stroke is caused by thrombotic or embolic occlusion of a cerebral artery [4, 5].

Currently, the main treatment for ischemic stroke could be summarized to three categories: mild hypothermia therapy [6, 7], thrombolysis [8–10] and mechanical thrombectomy [11, 12]. Thrombolysis is the only approbatory therapy for acute ischemic stroke in North America [13]. Nonetheless, it only takes effects in 3 hours. Beyond this span cerebral hemorrhage and edema are more risky. Although numerous potential treatment strategies have been investigated, most of them have been proven inefficacious in humans in a vigorous trial design [14, 15]. Therefore, there is an urgent demand to explore new or alternative anti-ischemic stroke agents.

**Fig 1:**
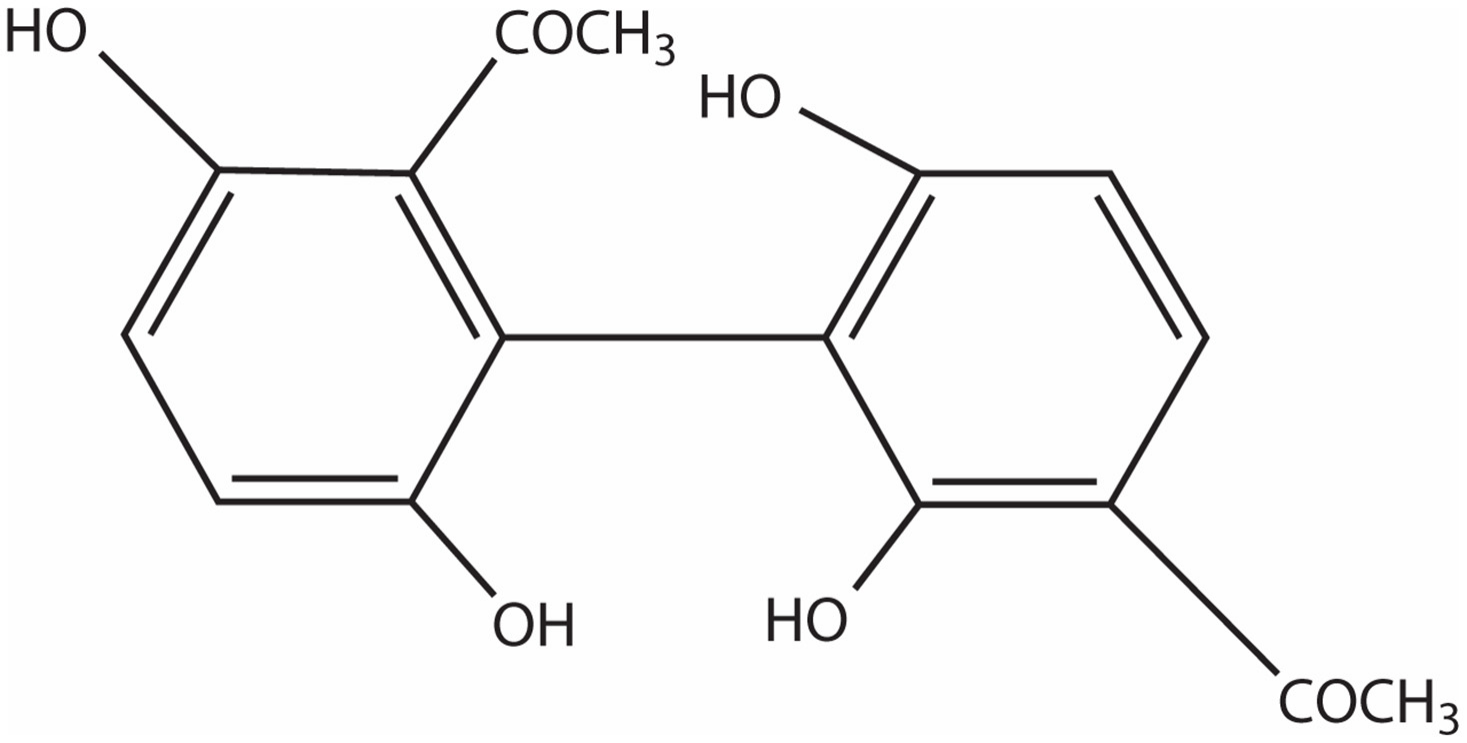
The chemical structure of CYNA.

*Cynanchum bungei* is a species of Polygonum multiflorum. As the Apocynaceae Cynanchum plants, it is widely distributed in China. CYNA, an acetophenone molecule, is a main component from the root of *Cynanchum bungei* and other species. Fig 1 shows its chemical structure. Earlier studies have demonstrated that CYNA possesses neuroprotective and hepatoprotectives effects [16, 17].

A series of experimental studies by one of our laboratories have been conducted on CYNA’s effects on ischemic stroke in rats. Evidence has shown that CYNA manifests itself as effective antioxidant vigor and behaves neuroprotectively both *in vitro* and in a rat model of transient focal cerebral ischemia with diminished neurological scarcity scores and infarct size [18]. These phenomena suggested that CYNA be further exploited as a therapy to protect nerves in ischemic stroke treatment. On the other hand, it has been realized that network based analysis is suitable for understanding the action mode of natural compounds which exhibit low affinity inhibition on multiple targets [19–21].

In this paper, we studied the anti-ischemic stroke effects of CYNA from two aspects, pathways and PPI networks. We first collected genes associated with ischemic stroke and putative targets of CYNA. Then, in order to identify pathways significantly regulated by CYNA, we constructed pathway sub-networks and scored the impact of CYNA on these sub-networks. At last, the network based anti-ischemic stroke effect score was defined based on the random walk with restart to quantitatively analyze the anti-ischemic stroke effect of CYNA. Experiments were also carried out in PC12 cells and cerebellar granule neurons to confirm the neuroprotective effects of CYNA *in vivo* and validate two targets of CYNA identified by our comparative proteomic experiment.

## Materials and Methods

### Experimental tests and validation

#### Reagents

Dulbecco’s Modified Eagle’s Medium (DMEM) and fetal bovine serum (FBS) were purchased from Gibco (Grand Island, NY). Antibodies for KHSRP (#13398), HMGB1 (#6893), and β-actin were obtained from Cell Signaling Technology (Beverly, MA).

#### Cells cultures and drug treatments

The high differentiated rat pheochromocytoma tumor cell line PC12 (Cell Bank of the Shanghai Institute of Biochemistry & Cell Biology, Shanghai Institute for Biological Sciences, Chinese Academy of Sciences, Shanghai, China, http://www.cellbank.org.cn) was maintained in DMEM containing 10% heat-inactivated equine serum and 5% FBS supplemented with 100 U/mL penicillin and 100 μg/mL streptomycin in a humidified atmosphere containing 5% CO_2_ at 37°C. For neuronal cultures, primary cultures of mouse (C57BL/6) cerebellar granule neurons were prepared from 6- to 8-day-old postnatal mice (Shanghai SLAC Lab. Animal Co., Ltd.). Briefly, cerebella were dissected after decapitation and cleaned free of meninges, and then treated with 0.025% w/v trypsin solution for 30 min at 37°C. A trypsin inhibitor was then added to block the enzyme, and 0.05% w/v DNase was added to break DNAs from dead cells. After a series of trituration and mild centrifugation steps, the cells were plating in DMEM and 10% FBS supplemented with KCI to a final concentration of 30 mM. Cells were plated onto 24-well dishes containing poly-L-lysine-coated coverslips at a density of 6 × 10^5^ per well for 7 days. CYNA was dissolved in dimethyl sulfoxide (DMSO) (Sigma. St. Louis, MO) and were freshly prepared each time before use (DMSO final culture concentration < 0.1%). For neuroprotection assay, cells were treated with 1, 10 and 100 μM CYNA for 6 h before exposure to 5 mM glutamate for PC12 cells or 100 μM glutamate for cerebellar granule neurons and then maintained for 24 h. At the end of the treatment period, protein lysates were prepared and western blot analysis was performed.

The study protocol was approved by the local institutional review board at the authors’ affiliated institutions and animal experiments were carried out in accordance with the established institutional guidelines for animal care and use at the Second Military Medical University.

#### Cell viability assays

Cell viability was measured by the 2-(2-methoxy-4-nitrophenyl)-3-(4-nitrophenyl)-5-(2,4-Disulfophen-yl)-2H-tetrazolium monosodium salt method using Cell Counting Kit-8 (Dojindo, Kumamoto, Japan) according to the manufacturer’s instructions. Absorbance was read at 450 nm by a BioTek Synergy 2 plate reader (BioTek Instruments, Inc., Winooski, Vt, USA).

#### Western blotting

Cells were collected by centrifugation at 1000 g for 3 min. The cell pellets were then washed with PBS, resuspended in lysis buffer containing 150 mM NaCl, 50 mM Tris (pH 8.0), 0.02% NaN_3_, 0.01% PMSF, 0.2% aprotinin, 1% tritonX-100 supplemented with protease inhibitor cocktail (Thermo Scientific), and centrifuged at 12,000 g for 10 min. The concentration of total proteins was determined by a BCA kit (Pierce, Rockford, IL). 30 μg proteins per lane was electrophoresed on 10% SDS polyacrylamide gels after boiling for 5 min and transferred to nitrocellulose membranes. Nonspecific reactivity was blocked by 5% nonfat milk prepared in TBST (10 mM Tris, 150 mM NaCl, 0.05% Tween-20, pH 7.5) at room temperature for 1 h. The membranes were incubated with antibodies diluted according to the manufacturers' instructions. Blots were washed three times in TBST, followed by incubation with the appropriate horseradish peroxidase-linked secondary antibodies for 1 h at room temperature. The proteins in the blots were visualized using the ECL plus system (Amersham Pharmacia Biotech, Buckinghamshire UK).

#### Statistical Analysis

Data were analyzed using GraphPad Prism software version 5 (Graph Pad software Inc., San Diego, CA, USA). Multiple comparisons were compared by one-way ANOVA analysis of variance followed by Tukey post hoc test. The quantitative data were reported as means ± SEM from at least three independent experiments. Statistical significance was determined as P < 0.05.

### Data preparation

#### Ischemic stroke associated genes

The ischemic stroke associated genes were collected from databases OMIM (The Online Mendelian Inheritance in Man database) and GAD (Genetic Association Database).

OMIM[22] is a database concerning human gene and genetic disarrays. It classifies all the known diseases with a genetic component and connects them to the interrelated genes in the human genome, with text information and reference information, sequence records, human genome and other data contained. With the keyword “Ischemic stroke”, we searched the OMIM database and found 5 associated genes, *ALOX5AP*, *F2*, *F5*, *NOS3* and *PRKCH*.

GAD[23] is a database of human genetic league researches of complicated diseases. It embraces brief data distilled from published papers in peer reviewed journals on candidate gene and GWAS researches. With the same keyword, we searched the GAD and found 60 genes whose association with ischemic stroke was not “N”.

Based on the above two databases, 61 distinct ischemic stroke associated genes were obtained. Among them, four genes in the OMIM database are also contained in the GAD, which are *ALOX5AP*, *F2*, *F5* and *NOS3*. The detail information could be seen in S1 Table.

#### Putative targets for CYNA

We obtained 17 putative targets of CYNA using two methods as follows.

Comparative proteomic analysis: In our earlier study, we carried out comparative proteomic analysis by matrix-assisted laser desorption/ionization-time of flight (MALDI-TOF) MS/MS of the pheochromocytoma tumor cell line PC12 cells treated with CYNA[18].

Here we briefly describe the process of the matrix-assisted laser desorption/ ionization-time of flight (MALDI-TOF) MS/MS. Each sample was suspended in 0.7 μL matrix solution containing a-cyano-4-hydroxycinnamic acid in acetonitrile/water (1:1, v/v) acidified with 0.1% trifluoroacetic acid. Then the mixture was immediately spotted onto the MALDI target. Analyses were performed on a 4700 Proteomics Analyzer equipped with a 355 nm Nd:YAG laser. The proteins were identified by peptide mass fingerprinting and tandem mass spectrometry using the program MASCOT v. 1.9 (Matrix Science, London, UK) against the SWISS-PROT database with the GPS explorer software (Applied Biosystems). MASCOT protein scores (based on combined MS and MS/MS spectra) of greater than 64 were considered statistically significant (*P* < 0.05).

The experiment identified 11 differentially expressed proteins in PC12 cells caused by the treatment of 10*μM* CYNA, in which only one protein (heterogeneous nuclear ribonucleoprotein *H1HNRNPH1*) was up-regulated. We considered this protein as a side-effect target and excluded it from our study. We mapped the 10 down-regulated rat proteins to human genome by homologous analysis and got 10 putative protein targets of CYNA. See Table 1 for detail information.

**Table 1:** The CYNA's targets obtained from comparative proteomic analysis, where *p_t_* and *p*_*t*, *comp*_ represent a rough estimate of the amount of pulled-down protein before and after treatment by CYNA, respectively.

Spot no.	NCBI accession no.	Homologous hunam gene	Entrez ID	*p*_*t*_	*p*_*t*,*comp*_	*Ratio*
1	gi|71043680	TUBB6	84617	29.03	18	0.62
2	gi|74271863	SARNP	84324	10.64	5	0.47
3	gi|149025908	TAF13	6884	6.82	3	0.44
4	gi|149048131	LMNA	4000	116.94	36.25	0.31
5	gi|149048132	EIF6	3692	127.08	30.5	0.24
6	gi|149028151	KHSRP	8570	56.098	23	0.41
7	gi|40018566	IMPDH2	3615	50.00	21	0.42
8	gi|6754208	HMGB1	3146	23.40	11	0.47
**9**	**gi|10946928**	**HNRNPH1**	3187	**8.09**	**19**	**2.35**
10	gi|40254595	DPYSL2	1808	62.50	20	0.32
11	gi|203278	CLTC	1213	24.24	8	0.33

Similarity search method: We used chemical similarity search tool to screen similar drugs of CYNA through the structural similarity comparison with the condition that the similar score is higher than 0.8 from TTD[24] (therapeutic target database). Totally, we obtained 2 similar drugs and 8 target proteins. See Table 2 for detail.

**Table 2:** CYNA's targets obtained from similarity search method.

Similarity drug(Tanimoto Coefficient)	Target gene	Entrez ID
Tolcapone(0.86)	FLT3 FLK1	2322
	KIT	3815
	PDGFRA	5156
Masoprocol(0.83)	KDR KDR	3791
	PRKCB PKCB PRKCB1	5579
	PRKCA PKCA	5578
	PRKACA	5566
	PRKCQ PRKCT	5588

#### Protein-Protein Interaction Data

Protein-protein interactions between human proteins were downloaded from the version 9.05 of STRING[25]. STRING includes physical and operational interplays congregated from numerous sources, including experimental archives, computational forecast algorithms, and public text collections. An evaluation system is used to weigh the evidence of each interaction. The interaction scores were normalized to the interval [0, 1]. It contains 16886 nodes and 1520927 edges.

#### Data of pathway gene sets and construction of pathway sub-networks

The pathway gene sets were downloaded from the C2: CP collection of MSigDB[26] database which were curated from several online pathway databases including bioCarta[27], KEGG[28], reactome[29] and so on. A total of 4722 pathways were included in this collection.

Then for each pathway gene set, we mapped its genes to the human PPI network and extracted the sub-network including all the genes and their interactions. In this way, we obtained all the pathway sub-networks for the CP collection of MSigDB database. We can see that a pathway sub-network is a connected fraction of the human protein-protein interaction network, in which all the genes perform the same cell function

### Scoring the impact of CYNA on the pathway sub-network

Recent study found that a pathway sub-network can be impacted by drug’s targets through the following two ways [30]:

1. A node of the pathway sub-network is acted on by a drug directly.
2. A periphery node of the pathway sub-network, which interacts with the pathway sub-network, is acted on. This case also should be included in our analysis.

We apply the score *s* to weigh how strong a pathway sub-network is affected by CYNA. The *s*-score is defined by the combination of different features of the pathway sub-network as follows [30]:

$$s=\frac{n_{dis,net}}{n_{net}}\times \frac{n_{tar,net}}{n_{net}}\times \frac{\sum_{t\in T_{t\mathrm{ar},net}}a_{t}}{\sum_{t\in T_{t\mathrm{ar}}}a_{t}}$$ (1)

Where *n*_*net*_ denotes the number of genes on the pathway sub-network, *n*_*dis*,*net*_ denotes the number of ischemic stroke associated genes on the pathway sub-network. Hence ndis,netnnet represents the ratio of ischemic stroke associated genes to the total size of the affected pathway sub-network, i.e., how frequently genes of this disease are present in the sub-network. Similarly, *n*_*tar*,*net*_ is the number of CYNA's targets on the pathway sub-network and its periphery nodes, while ntar,netnnet puts the impact of CYNA in relation to the size of the sub-network. Besides the number of the target on the pathway sub-network, the affected strength of CYNA to ischemic stroke, i.e., binding affinity also should be considered [31]. The affinity measure is derived from chemical proteomics data directly and obtained by the following equation:

$$a_{t}=(1-\frac{p_{t,comp}}{p_{t}})\times \ln(p_{t})$$ (2)

Where *p*_*t*_ and *p*_*t*,*comp*_ represent a rough estimate of the amount of pulled-down protein before and after treatment by CYNA, respectively. Since in chemical proteomics the drug is always presented at a large excess of constant concentration, ln(*p*_*t*_) is used to down weigh parameter influence. Therefore, $\sum_{t\in T_{t\mathrm{ar}}}a_{t}$ and $\sum_{t\in T_{t\mathrm{ar},net}}a_{t}$ denote the sum of affinities for the CYNA’s targets and targets on the pathway sub-network and its periphery nodes, respectively. Hence, the last feature ∑t∈Ttar,netat∑t∈Ttarat could be interpreted as the ratio of CYNA’s affinities on the pathway sub-network to overall affinity of CYNA’s targets used from comparative proteomic analysis.

### Network scoring anti-ischemic stroke of CYNA

#### Scoring network effect of a group of seed nodes

In order to obtain CYNA’s effect to all the genes on the PPI network, we applied the algorithm of random walk with restart, which is used in many areas, such as identifying of functional modules, modeling the evolution of social networks and so on [32,33]. The algorithm can compute all the nodes’ score of the network based on a group of seed nodes. In this paper, we used a weighted PPI network as the network and ischemic stroke associated genes or protein targets of CYNA as the seed nodes.

The algorithm could be described as follows: A seed node is chosen from the seed set *S* before the random walk starting.

At each step, the random walker either moves to a chosen neighbor *u*∊*N* of the current node *v*, randomly, or it restarts at one of the nodes in the seed set *S*. The probability of restarting at a given time step is a fixed parameter, which is denoted by *r*. For each restart, the probability of restarting at *v*∊*S* suggests the degree of association between *v* and the seed set *S*. For each move, the probability of moving to interacting partner *u* of the current node *v* is proportional to the reliability of the interaction between *u* and *v*. This process could be represented as follows:

$$x^{t+1}=(1-r)Px^{t}+rx^{0}$$ (3)

where P is the adjacency matrix of the weighted PPI network, representing the coupling strength of nodes in the network; *r* ∊ [o,1] is a parameter denoting the restart probability which needs to be calibrated with real data; *x* ^*t*^ is a vector in which *x* ^*t*^(*v*) denotes the probability that the node will be at node *v* at time *t*; *x* ^*0*^ is a vector representing the strength of seed nodes. After a sufficiently long time, the probability of being at node *v* at a random time step provides a measure of the functional association between *v* and the genes in seed set *S*, hence, the effect strength of seed set S to each nodes in the network is defined by steady-state probability vector *x* ^∞^ when *x* ^*t*+1^ = *x* ^*t*^.

#### Scoring ischemic stroke’s effect on the human PPI network

Taking ischemic stroke associated genes as the seed nodes. Although it can be assumed that the initial strength values *x*_0_(*v*) of different seed nodes are different as the associated degree of different ischemic stroke genes to ischemic stroke is varying, for simplicity, all ischemic stroke associated genes are treated equally in this algorithm, and initial vector *x*_0_ could thus be defined as *x*_0_(*v*) = 1 if v is a seed otherwise *x*_0_(*v*) = 0.

Then ischemic stroke effect score of each node in the human network was computed by random walk with restart and an ischemic stroke’s effect vector x_is_ was obtained.

#### Scoring CYNA’s effect on the human PPI network

In this case, CYNA’s effect on the human PPI network is studied. The seed nodes are defined as CYNA’s targets. Similarly, the effected strength of the CYNA’s targets to the ischemic stroke is set as the initial strength values *x*_0_(*v*) of seed nodes. The affinities of CYNA’s targets obtained from comparative proteomic experiment are known, and could be used to define initial strength value of a seed node [34].

For CYNA, its effect score on each node in the human network was computed by random walk with restart and its drug effect vector *x*_*ca*_ was obtained.

#### Scoring the anti-ischemic stroke effects of CYNA

The inner product between the vectors of disease effect and CYNA effect was applied to measure how CYNA impacts the human interactome under the influence of ischemic stroke [34]. In this paper, *E* = < *x*_*is*,_ *x*_*ca*_ >is defined as the anti- ischemic stroke effect score of CYNA. The effect score of CYNA was then compared with that of its random contracts by Z-score.

## Results and Discussion

### CYNA protected against glutamate-induced neurotoxicity in PC12 cells and cerebellar granule neurons

**Fig 2:**
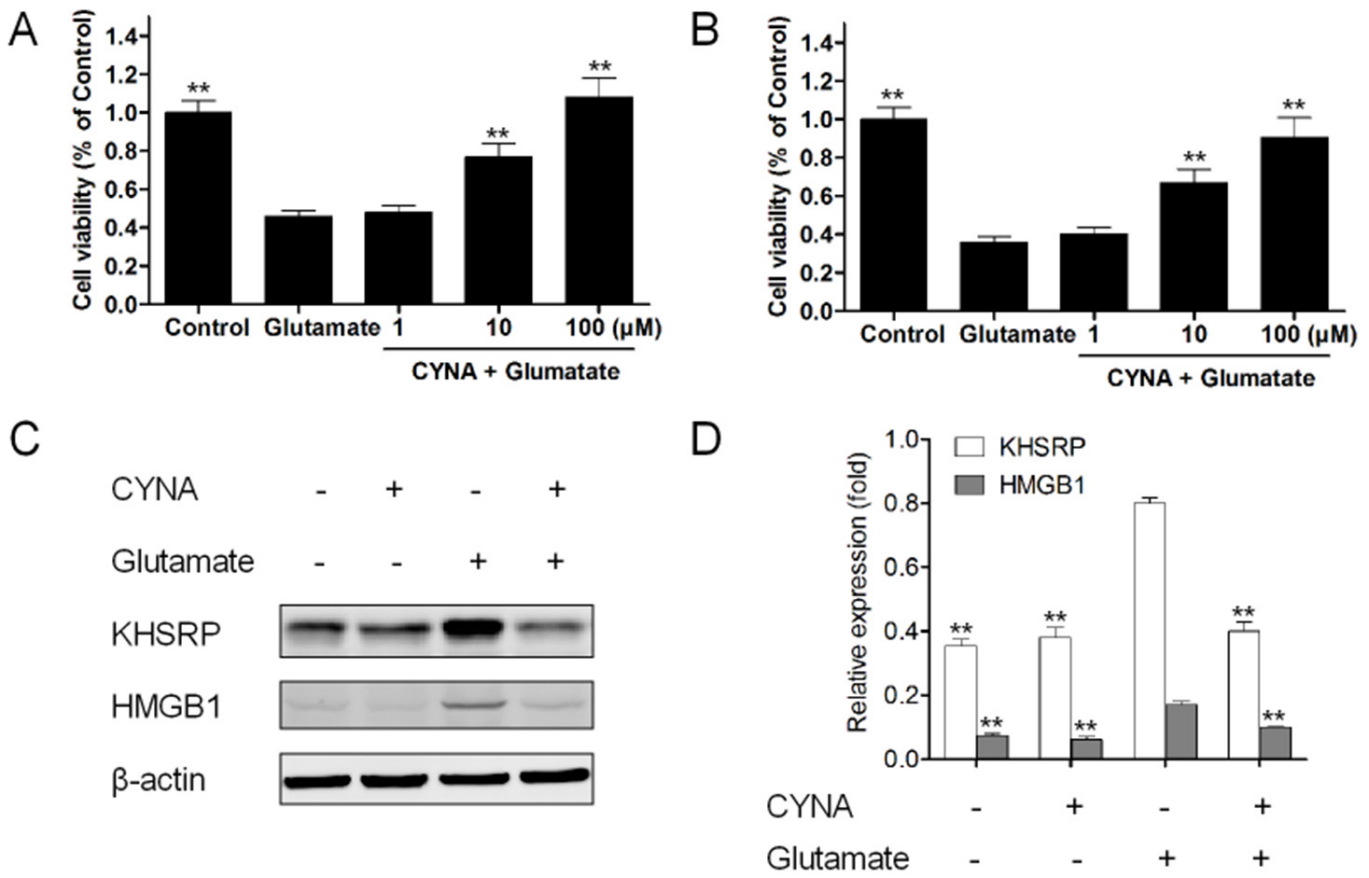
CYNA protected against glutamate-induced neurotoxicity in PC12 cells and cerebellar granule neurons. (A) Effects of CYNA on 5 mM glutamate-induced PC12 cell injury. (B) Effects of CYNA on 100 μM glutamate-induced cerebellar granule neurons. Cells were treated with 1, 10, and 100 μM CYNA and then co-incubated with or without glutamate for 24 h, and cell viability was determined by CCK8 assay. Data are presented as mean ± SEM (n = 6). ** P < 0.01 versus glutamate by one-way ANOVA analysis of variance with Tukey’s HSD post hoc test. (C) Proteins extracted from glutamate-treated cerebellar granule neurons either with or without pre-incubation with CYNA were subjected to western blotting to detect changes in KHSRP and HMGB1 expression. 10 μM CYNA prevented the upregulation of KHSRP and HMGB1 after 100 μM glutamate-treated. (D) Data shown are the results of three different experiments and are represented as the relative densities of protein bands normalized to β-actin. Results are presented as means ± SEM of three assays. ** Significant difference compared with glutamate by one-way ANOVA analysis of variance with Tukey’s HSD post hoc test (*P* < 0.01).

We first validated whether CYNA could protect against oxidative glutamate cytotoxicity in PC12 cells and cerebellar granule neurons. As shown in Fig 2A, CYNA could dose-dependently mitigate 5 mM glutamate-induced neurotoxicity from 10 to 100 μM in PC12 cells. Similar findings were also obtained in cerebellar granule neurons exposed to 100 μM glutamate from 10 to 100 μM (Fig 2B).

Then, we verified CYNA’s impact on KHSRP and HMGB1, two targets of CYNA identified by our earlier comparative proteomic analysis (see Table 1) whose antibodies are commercially available. Proteins extracted from glutamate-treated cerebellar granule neurons either with or without pre-incubation with CYNA were subjected to western blotting study to investigate the changes of KHSRP and HMGB1’s expression (Fig 2C). 10 μM CYNA significantly prevented the upregulation of KHSRP and HMGB1 after 100 μM glutamate-treated (Fig 2D).

Together, these results confirmed that CYNA protected against glutamate-induced neurotoxicity in PC12 cells and cerebellar granule neurons and inhibited glutamate-induced upregulation of KHSRP and HMGB1.

### The impact of CYNA on the pathway sub-networks

We further extracted data related to ischemic stroke’s pathogenesis and treatment—ischemic stroke-associated genes and CYNA’s targets to demonstrate its anti-ischemic stroke effect on the pathway sub-networks. For each pathway sub-network, ischemic stroke associated genes and CYNA target genes on this pathway and its periphery were obtained. And the target profile is weighted with respect to its affinity. In order to remove the impact of network itself, we only studied the pathway sub-networks whose number of ischemic stroke associated gene is more than 3 and the number of CYNA's target genes is more than 2. A total of 182 pathway sub-networks satisfied these criteria. Then, all affected pathway sub-networks are scored using Eq (1), See S2 Table for detail. Finally, 25 sub-networks were obtained whose s-scores are higher than 2 fold average value (average value is 0.0049). See Table 3 for the detail.

**Table 3:** Pathways whose sub-network score *s* is higher than 0.0098, where *GN* represents sub-network gene number, *DN* and *TN* represent disease gene number and target gene number on the sub-network, respectively.

Pathway	*GN*	*DN*	*TN*	*AS*	*S*
**P130CAS LINKAGE TO MAPK SIGNALING FOR INTEGRINS**	**23**	**5**	**6**	**5.6394**	**0.0539**
**GRB2 SOS PROVIDES LINKAGE TO MAPK SIGNALING FOR INTERGRINS**	**23**	**5**	**6**	**5.6394**	**0.0539**
**FIBRINOLYSIS PATHWAY**	**19**	**6**	**4**	**4.6465**	**0.0521**
IL5 PATHWAY	17	4	4	4.6755	0.0436
**PLATELET ADHESION TO EXPOSED COLLAGEN**	**18**	**4**	**4**	**4.0232**	**0.0335**
HDAC TARGETS DN	22	4	5	4.7189	0.0329
**INTEGRIN ALPHAIIB BETA3 SIGNALING**	**35**	**5**	**6**	**5.6394**	**0.0233**
**LAIR PATHWAY**	**22**	**6**	**3**	**3.5715**	**0.0224**
**GRANULOCYTES PATHWAY**	**18**	**4**	**3**	**3.5715**	**0.0223**
**PLATELET AGGREGATION PLUG FORMATION**	**44**	**7**	**6**	**5.6394**	**0.0206**
RESPONSE TO TSA UP	37	4	7	5.9313	0.0205
**INTRINSIC PATHWAY**	**21**	**4**	**4**	**2.8489**	**0.0174**
AMB2 NEUTROPHILS PATHWAY	48	7	6	5.6394	0.0173
**UPA UPAR PATHWAY**	**48**	**7**	**6**	**5.6394**	**0.0173**
NFKB TARGETS REPRESSED BY GLUCOCORTICOIDS	31	5	4	4.6755	0.0164
**FORMATION OF FIBRIN CLOT CLOTTING CASCADE**	**36**	**9**	**4**	**3.3027**	**0.0155**
EPHRINBREVPATHWAY	36	4	5	5.0232	0.0131
LVAD SUPPORT OF FAILING HEART DN	38	4	5	5.2991	0.0124
INTEGRIN2 PATHWAY	36	5	4	4.6465	0.0121
TNF TARGETS	26	4	3	3.5715	0.0107
IL23PATHWAY	45	5	5	5.0232	0.0105
INFLAM PATHWAY	35	4	4	4.6755	0.0103
TYPE I DIABETES MELLITUS	43	6	4	4.6538	0.0102
INTEGRIN3 PATHWAY	48	4	6	5.6394	0.0099
LRRC3B TARGETS	37	4	4	4.9514	0.0098

*AS* represents affinity score. Blackbodies represent the pathways associated with the biological process of thrombotic or embolic occlusion of a cerebral artery.

As shown in Table 3, there are 25 pathways whose scores are more significant than the others. Among them, the score of pathways *P130CAS LINKAGE TO MAPK SIGNALING FOR INTEGRINS* and *GRB2 SOS PROVIDES LINKAGE TO MAPK SIGNALING FOR INTERGRINS* even reach 0.0539.

It is found that 11 of the 25 pathways are associated with the biological process of thrombotic or embolic occlusion of a cerebral artery, which is the main cause of ischemic stroke. Specifically, the pathways *P130CAS LINKAGE TO MAPK SIGNALING FOR INTEGRINS and GRB2 SOS PROVIDES LINKAGE TO MAPK SIGNALING FOR INTERGRINS* play the same role, in which the platelets were stimuli activated by bioactive molecules such as thrombin, ADP, collagen, fibrinogen and thrombospondin, and the activated platelet integrin alphaIIbbeta3 interacts with the fibrinogen and links platelets together in an aggregate to form a platelet plug [35–40]. In the process of *FIBRINOLYSIS PATHWAY*, overabundance or increased activity of the plamsminogen activator inhibitors or reduced presence or function of tissue-type plasminogen activator (tPA) or urokinase plasminogen activator (uPA) can cause an increase in fibrin deposition or the formation of a thrombus, which further result in atherosclerotic disease and venous thrombosis [41,42]. As for the pathway of *PLATELET ADHESION TO EXPOSED COLLAGEN*, under acute vascular trauma, vasoconstrictor mechanisms predominate and the endothelium becomes prothrombotic, procoagulatory and proinflammatory in nature. The chief trigger for the change in endothelial function that leads to the formation of a haemostatic thrombus is the loss of the endothelial cell barrier between blood and extracellular matrix components [43,44]. The pathway of *INTEGRIN ALPHAIIB BETA3 SIGNALING* causes the initiation of platelet adhesion which could result in the platelet plug [45,46]. The other 6 pathways are also associated with platelet plug [47–52]. These results suggest that CYNA performs anti-ischemic stroke effect by regulating several biological processes associated with ischemic stroke.

**Fig 3:**
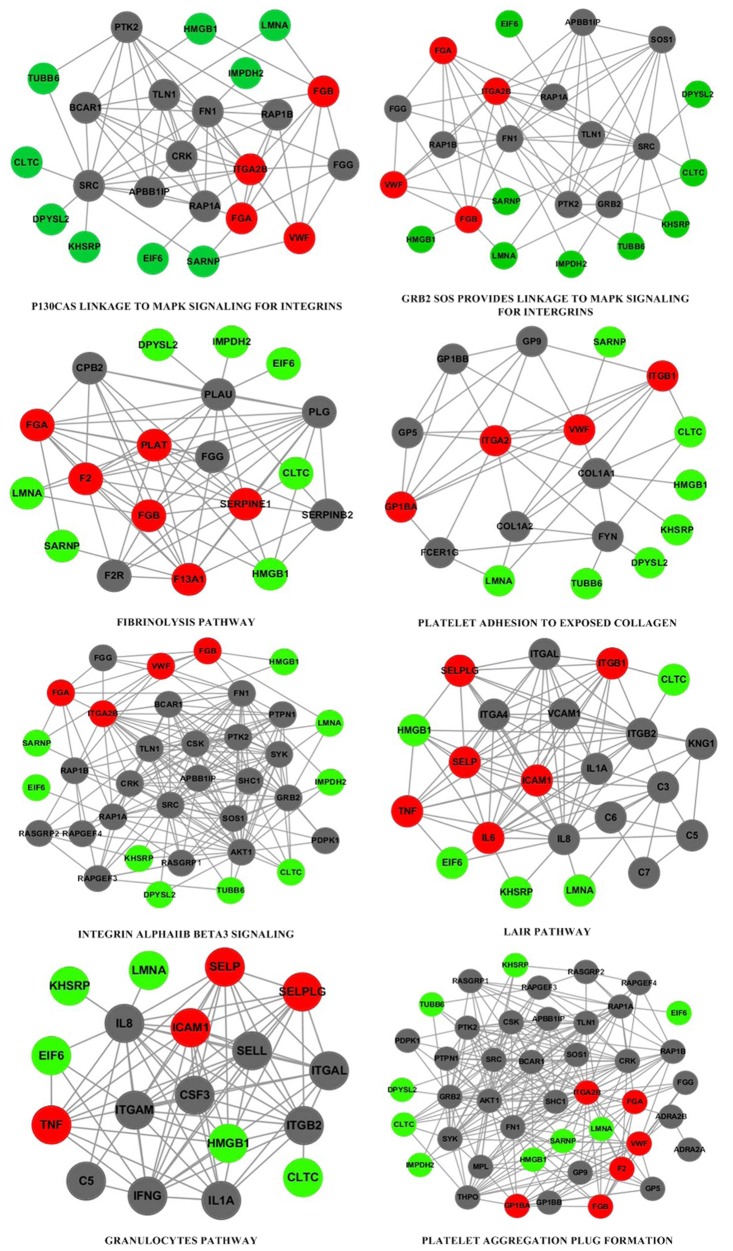
Eight ischemic stroke associated pathway sub-networks regulated by CYNA, where red circles represent ischemic stroke associated genes, green circles represent CYNA's targets, gray circles represent other genes on the pathway.

Fig 3 shows 8 of the 11 ischemic stroke associated pathway sub-networks. It can be seen that disease genes *VWF*, *F2*, *ITGB3*, *FGA* and *FGB* appear on most sub-networks, suggesting their important roles in ischemic stroke. Although the CYNA’s targets hit few nodes on these sub-networks, their periphery nodes contain most of CYNA’ targets, implying that the targets act on the pathways through interactions on PPI network.

On the other hand, other pathways activated or inhibited by CYNA are not directly correlated with ischemic stroke pathogenesis. Some of them have indirect relationship with the disease pathogenesis. Taken IL5 PATHWAY as an example, *IL-5* is an inflammatory signaling molecule that primarily stimulates eosinophil proliferation, maturation and activation [53]. Secreted IL-5 stimulates productions that migrate to tissues in response to eotaxin and release factors that damage tissues, causing some of the undesirable consequences of inflammation. Once inflammation infects one cerebral artery, it may cause thrombotic or embolic occlusion. Another example is *INTEGRIN2 PATHWAY* [54]. Integrins are cell surface receptors that interact with the extracellular matrix and mediate intracellular signals in response to the extracellular matrix including cellular shape, mobility, and progression through the cell cycle. Growth factor signaling pathways and the caveolin receptor exhibit important cross talk with integrin receptors in cellular responses like activation of map kinase, proliferation and motility. If some disorders occur in the process of *INTEGRIN2 PATHWAY*, blood vessel cell proliferation and motility may be destroyed and lead to thrombotic or embolic occlusion. Hence these pathways may give new suggestions to identify other targets in stroke.

### Anti-ischemic stroke effects of CYNA by network scores

#### Calculating the optimal r for the CYNA

**Fig 4:**
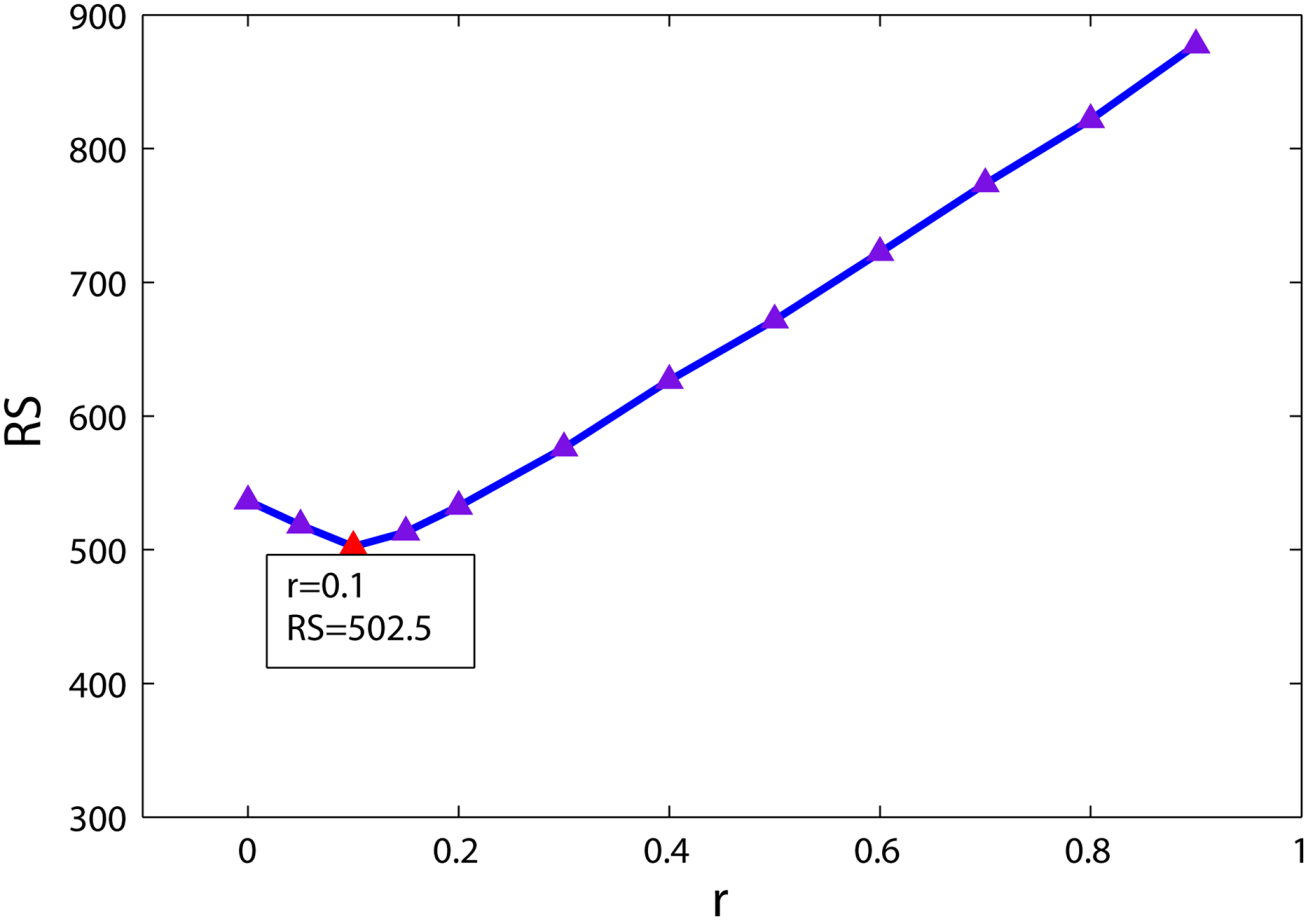
The curve of the correlation between *r* value and *RS* value, where triangle presents a *r* value for a *RS* value. Red triangle represents the optimal *r* and its responding *RS*.

The algorithm of random walk with restart has been successfully used in the prioritization of candidate disease genes and *r* = 0.3 appeared to be a robust choice [55]. Thus we took *r* = 0.3 to score ischemic stroke’s effect on the human PPI network in this study. Since *r* = 0.3 was got by fitting real data of disease genes, it may not be optimum for estimating the impact of the small molecule CYNA on the network. Therefore, we tried to find the optimal *r* value by the following procedure:

1. Defining targets obtained from comparative proteomic experiment as seed nodes, and then defining targets obtained from similarity search method as test set *P*;
2. Taking *r* = 0 and calculating the score for the anti-ischemic stroke effects of CYNA on the human PPI network by Eq (3);
3. Descendingly ranking the genes according their scores;
4. Calculating the average ranking score *RS* of genes in the test set P;
5. Seting *r* = *r*+0.05, and using the above procedure to obtain the corresponding *RS* value;

Continuing to implement the above procedure, we use different *r* values to obtain the corresponding *RS* values. Fig 4 shows the relationship between *r* and *RS*. It can be see that the curve of the correlation between *r* value and *RS* value is a notching curve. *RS* value decreases first and then increases. It reaches minimum when *r* = 0.1. Therefore, the optimal *r* value for CYNA was taken as 0.1.

#### Network scoring anti-ischemic stroke of CYNA

The network score was computed in order to explain the anti-ischemic stroke effect of CYNA quantitatively. The targets obtained from comparative proteomics experiment and similarity search method were combined as the group of seed nodes (17 nodes). As a naturally-occurring substance, inhibition potency of CYNA on targets could be much weaker, unlike that of specifically designed drug molecules. Therefore, we defined the components of the initial vector *x*_0_ corresponding to targets of CYNA obtained by comparative proteomics experiment as the normalized *a*_*t*_, and the components of the initial vector *x*_0_ corresponding to targets of CYNA by similarity search method as the average of all the normalized *a*_*t*_ value, otherwise *x*_0_(*v*) = 0.

Score vector *x*_*is*_ of ischemic stroke’s effect on the human PPI network and score vector *x*_*ca*_ of the anti-ischemic stroke effects of CYNA were respectively obtained based on Eq (3) and associated data. Then CYNA’s effect on the human PPI network was calculated by *E* = < *x*_*is*_, *x*_*ca*_ >. The effect score is 0.0589.

Then 1000 random target sets, containing same number of proteins as CYNA’s targets, were generated to find out whether CYNA's effect score suggests significant anti-ischemic stroke effect. The mean effect score and the standard deviation of the 1000 random counterparts were calculated, and the z-score of CYNA’s anti-ischemic stroke effect score was obtained. The absolute value of z-score greater than 3 indicates a statistically significant deviation between the actual value and the random ones. Thus the z-score 3.803 of CYNA suggests its significant anti-ischemic stroke effect. In fact, earlier study has reported the effects of CYNA on ischemic stroke [15, 18, 56–58].

## Conclusions

This study demonstrates the anti-ischemic stroke effect of CYNA from a network perspective.

First, we validated the neuroprotective effects of CYNA and found that it could protect against glutamate-induced neurotoxicity in PC12 cells and cerebellar granule neurons. Furthermore, we have extracted data related to ischemic stroke’s pathogenesis and treatment—ischemic stroke-associated genes from various databases and CYNA’s targets, respectively. Then two network methods were applied to illustrate CYNA’s effect to ischemic stroke. On the pathway sub-networks, each score of the impact of CYNA to ischemic stroke was obtained. Half of the high score pathways were associated with the biological process of thrombotic or embolic occlusion of a cerebral artery, which is the main cause of ischemic stroke. In addition, we also quantitatively analyzed the anti-ischemic stroke effect of CYNA. We got the anti-ischemic stroke effect score of CYNA as 0.0589, which is significantly higher than that of its random counterparts, suggesting significant anti-ischemic stroke effect of CYNA. This work applied network approach to explain CYNA’s anti-ischemic stroke effect from two aspects, respectively, which may give an inspiration to study complex diseases’ pathogenesis and treatments.

## Supporting Information

S1 Table Genes associated with ischemic stroke from two resources. (DOCX)

S2 Table The score of the impact of CYNA on the pathway sub-network. (DOCX)
