# Supplementary material for: Analysis of Cynandione A’s Anti-Ischemic Stroke Effects from Pathways and Protein-Protein Interactome
Source: PLoS One. 2015 May 8;10(5):e0124632. doi: 10.1371/journal.pone.0124632 (PMC4425521; doi:10.1371/journal.pone.0124632)
Supplement: S1 Table — (DOCX) [file pone.0124632.s001.docx]

**Supplementary file for “****Analysis of** **cynandione A’s anti-** **ischemic stroke effects from pathways and protein-protein interactome”**

## S1 Table. Genes associated with ischemic stroke from two resources

| **Gene name** | **ID** | **Database** |
| --- | --- | --- |
|  |  |  |
| ACE | 1636 | GAD |
| ADAMTS13 | 11093 | GAD |
| ADIPOQ | 9370 | GAD |
| AGER | 177 | GAD |
| ALOX5AP | 241 | GAD OMIM |
| APOA5 | 116519 | GAD |
| APOE | 348 | GAD |
| CAPN10 | 11132 | GAD |
| CCL2 | 6347 | GAD |
| CD14 | 929 | GAD |
| CRP | 1401 | GAD |
| EPHX2 | 2053 | GAD |
| F12 | 2161 | GAD |
| F13A1 | 2162 | GAD |
| F2 | 2147 | GAD OMIM |
| F5 | 2153 | GAD OMIM |
| F7 | 2155 | GAD |
| FGA | 2243 | GAD |
| FGB | 2244 | GAD |
| GCLM | 2730 | GAD |
| GP1BA | 2811 | GAD |
| GPX3 | 2878 | GAD |
| HIF1A | 3091 | GAD |
| HLA-B | 3106 | GAD |
| HLA-DQB1 | 3119 | GAD |
| HLA-DRA | 3122 | GAD |
| HLA-DRB1 | 3123 | GAD |
| HSPA1A | 3303 | GAD |
| HSPA1B | 3304 | GAD |
| HSPA1L | 3305 | GAD |
| ICAM1 | 3383 | GAD |
| IL1B | 3553 | GAD |
| IL6 | 3569 | GAD |
| ITGA2 | 3673 | GAD |
| ITGA2B | 3674 | GAD |
| ITGA3 | 3675 | GAD |
| ITGB1 | 3688 | GAD |
| ITGB3 | 3690 | GAD |
| KL | 9365 | GAD |
| MIF | 4282 | GAD |
| MMP3 | 4314 | GAD |
| MTHFR | 4524 | GAD |
| NOS3 | 4846 | GAD OMIM |
| NPR3 | 4883 | GAD |
| OLR1 | 4973 | GAD |
| PDE4D | 5144 | GAD |
| PLAT | 5327 | GAD |
| PON1 | 5444 | GAD |
| PON2 | 5445 | GAD |
| PRKCH | 5583 | OMIM |
| PROZ | 8858 | GAD |
| SELE | 6401 | GAD |
| SELP | 6403 | GAD |
| SELPLG | 6404 | GAD |
| SERPINA1 | 5265 | GAD |
| SERPINE1 | 5054 | GAD |
| SLC6A4 | 6532 | GAD |
| SLC9A1 | 6548 | GAD |
| TLR4 | 7099 | GAD |
| TNF | 7124 | GAD |
| VWF | 7450 | GAD |
