# Supplementary material for: Analysis of Cynandione A’s Anti-Ischemic Stroke Effects from Pathways and Protein-Protein Interactome
Source: PLoS One. 2015 May 8;10(5):e0124632. doi: 10.1371/journal.pone.0124632 (PMC4425521; doi:10.1371/journal.pone.0124632)
Supplement: S2 Table — (DOCX) [file pone.0124632.s002.docx]

**Supplementary file for “****Analysis of** **cynandione A’s anti-** **ischemic stroke effects from pathways and protein-protein interactome”**

## S2 Table. The score of the impact of cynandione A on the pathway sub-network

| **Pathway** | **GN** | **DN** | **TN** | **AS** | **s-score** |
| --- | --- | --- | --- | --- | --- |
| REACTOME_P130CAS_LINKAGE_TO_MAPK_SIGNALING_FOR_INTEGRINS | 23 | 5 | 6 | 5.6394 | 0.0539 |
| REACTOME_GRB2_SOS_PROVIDES_LINKAGE_TO_MAPK_SIGNALING_FOR_INTERGRINS_ | 23 | 5 | 6 | 5.6394 | 0.0539 |
| BIOCARTA_FIBRINOLYSIS_PATHWAY | 19 | 6 | 4 | 4.6465 | 0.0521 |
| BIOCARTA_IL5_PATHWAY | 17 | 4 | 4 | 4.6755 | 0.0436 |
| REACTOME_PLATELET_ADHESION_TO_EXPOSED_COLLAGEN | 18 | 4 | 4 | 4.0232 | 0.0335 |
| MARKS_HDAC_TARGETS_DN | 22 | 4 | 5 | 4.7189 | 0.0329 |
| REACTOME_INTEGRIN_ALPHAIIB_BETA3_SIGNALING | 35 | 5 | 6 | 5.6394 | 0.0233 |
| BIOCARTA_LAIR_PATHWAY | 22 | 6 | 3 | 3.5715 | 0.0224 |
| BIOCARTA_GRANULOCYTES_PATHWAY | 18 | 4 | 3 | 3.5715 | 0.0223 |
| REACTOME_PLATELET_AGGREGATION_PLUG_FORMATION | 44 | 7 | 6 | 5.6394 | 0.0206 |
| MOREIRA_RESPONSE_TO_TSA_UP | 37 | 4 | 7 | 5.9313 | 0.0205 |
| REACTOME_INTRINSIC_PATHWAY | 21 | 4 | 4 | 2.8489 | 0.0174 |
| PID_AMB2_NEUTROPHILS_PATHWAY | 48 | 7 | 6 | 5.6394 | 0.0173 |
| PID_UPA_UPAR_PATHWAY | 48 | 7 | 6 | 5.6394 | 0.0173 |
| DEBOSSCHER_NFKB_TARGETS_REPRESSED_BY_GLUCOCORTICOIDS | 31 | 5 | 4 | 4.6755 | 0.0164 |
| REACTOME_FORMATION_OF_FIBRIN_CLOT_CLOTTING_CASCADE | 36 | 9 | 4 | 3.3027 | 0.0155 |
| PID_EPHRINBREVPATHWAY | 36 | 4 | 5 | 5.0232 | 0.0131 |
| CHEN_LVAD_SUPPORT_OF_FAILING_HEART_DN | 38 | 4 | 5 | 5.2991 | 0.0124 |
| PID_INTEGRIN2_PATHWAY | 36 | 5 | 4 | 4.6465 | 0.0121 |
| WANG_TNF_TARGETS | 26 | 4 | 3 | 3.5715 | 0.0107 |
| PID_IL23PATHWAY | 45 | 5 | 5 | 5.0232 | 0.0105 |
| BIOCARTA_INFLAM_PATHWAY | 35 | 4 | 4 | 4.6755 | 0.0103 |
| KEGG_TYPE_I_DIABETES_MELLITUS | 43 | 6 | 4 | 4.6538 | 0.0102 |
| PID_INTEGRIN3_PATHWAY | 48 | 4 | 6 | 5.6394 | 0.0099 |
| KIM_LRRC3B_TARGETS | 37 | 4 | 4 | 4.9514 | 0.0098 |
| KEGG_GRAFT_VERSUS_HOST_DISEASE | 41 | 7 | 3 | 4.3352 | 0.0091 |
| KEGG_COMPLEMENT_AND_COAGULATION_CASCADES | 77 | 11 | 5 | 4.9941 | 0.0078 |
| REACTOME_RESPONSE_TO_ELEVATED_PLATELET_CYTOSOLIC_CA2_ | 87 | 10 | 6 | 5.6394 | 0.0075 |
| BROWNE_HCMV_INFECTION_2HR_DN | 49 | 4 | 5 | 5.2991 | 0.0074 |
| REACTOME_INTEGRIN_CELL_SURFACE_INTERACTIONS | 85 | 9 | 6 | 5.6394 | 0.0071 |
| KEGG_ASTHMA | 32 | 4 | 3 | 3.2666 | 0.0065 |
| PID_INTEGRIN1_PATHWAY | 72 | 7 | 5 | 5.2918 | 0.0060 |
| KEGG_HEMATOPOIETIC_CELL_LINEAGE | 92 | 11 | 5 | 5.2918 | 0.0058 |
| KEGG_ANTIGEN_PROCESSING_AND_PRESENTATION | 83 | 7 | 6 | 5.6394 | 0.0058 |
| LINDSTEDT_DENDRITIC_CELL_MATURATION_A | 69 | 6 | 5 | 5.2991 | 0.0056 |
| PID_TXA2PATHWAY | 64 | 4 | 6 | 5.6394 | 0.0056 |
| DAUER_STAT3_TARGETS_UP | 51 | 4 | 5 | 4.2595 | 0.0055 |
| CORRE_MULTIPLE_MYELOMA_UP | 49 | 6 | 3 | 4.3061 | 0.0054 |
| PID_ANGIOPOIETINRECEPTOR_PATHWAY | 58 | 4 | 5 | 5.2991 | 0.0053 |
| DASU_IL6_SIGNALING_UP | 58 | 4 | 5 | 5.2991 | 0.0053 |
| HINATA_NFKB_TARGETS_FIBROBLAST_UP | 87 | 7 | 6 | 5.5910 | 0.0052 |
| WUNDER_INFLAMMATORY_RESPONSE_AND_CHOLESTEROL_UP | 52 | 5 | 4 | 4.1878 | 0.0052 |
| LEE_AGING_CEREBELLUM_UP | 84 | 5 | 7 | 5.9313 | 0.0050 |
| COATES_MACROPHAGE_M1_VS_M2_DN | 49 | 4 | 4 | 4.2918 | 0.0048 |
| HINATA_NFKB_TARGETS_KERATINOCYTE_UP | 98 | 8 | 6 | 5.5910 | 0.0047 |
| KEGG_ECM_RECEPTOR_INTERACTION | 88 | 7 | 6 | 4.8757 | 0.0045 |
| KEGG_ARRHYTHMOGENIC_RIGHT_VENTRICULAR_CARDIOMYOPATHY_ARVC | 80 | 5 | 6 | 5.6394 | 0.0045 |
| PETROVA_PROX1_TARGETS_DN | 65 | 4 | 5 | 5.2991 | 0.0042 |
| REACTOME_CELL_SURFACE_INTERACTIONS_AT_THE_VASCULAR_WALL | 90 | 6 | 6 | 5.6394 | 0.0042 |
| TENEDINI_MEGAKARYOCYTE_MARKERS | 73 | 5 | 5 | 5.2991 | 0.0042 |
| SANA_TNF_SIGNALING_DN | 83 | 4 | 7 | 5.9313 | 0.0041 |
| KEGG_VIRAL_MYOCARDITIS | 72 | 5 | 5 | 4.9748 | 0.0040 |
| LEIN_CHOROID_PLEXUS_MARKERS | 71 | 4 | 6 | 4.8757 | 0.0039 |
| KEGG_NOD_LIKE_RECEPTOR_SIGNALING_PATHWAY | 68 | 4 | 5 | 5.2991 | 0.0039 |
| VERRECCHIA_EARLY_RESPONSE_TO_TGFB1 | 64 | 5 | 4 | 4.6538 | 0.0038 |
| KEGG_AUTOIMMUNE_THYROID_DISEASE | 47 | 4 | 3 | 4.0303 | 0.0037 |
| KIM_GLIS2_TARGETS_UP | 87 | 6 | 5 | 5.2991 | 0.0035 |
| HARRIS_HYPOXIA | 91 | 4 | 7 | 5.9313 | 0.0034 |
| KEGG_INTESTINAL_IMMUNE_NETWORK_FOR_IGA_PRODUCTION | 51 | 4 | 3 | 4.3352 | 0.0034 |
| KEGG_HYPERTROPHIC_CARDIOMYOPATHY_HCM | 88 | 8 | 4 | 4.6828 | 0.0033 |
| KEGG_SMALL_CELL_LUNG_CANCER | 94 | 4 | 7 | 5.9313 | 0.0032 |
| CROMER_TUMORIGENESIS_UP | 65 | 4 | 4 | 4.9514 | 0.0032 |
| HELLEBREKERS_SILENCED_DURING_TUMOR_ANGIOGENESIS | 77 | 4 | 5 | 5.2991 | 0.0030 |
| KEGG_LEISHMANIA_INFECTION | 73 | 7 | 3 | 4.3352 | 0.0029 |
| GU_PDEF_TARGETS_UP | 79 | 4 | 5 | 5.2991 | 0.0029 |
| ICHIBA_GRAFT_VERSUS_HOST_DISEASE_D7_UP | 99 | 6 | 5 | 5.2991 | 0.0027 |
| MARKEY_RB1_CHRONIC_LOF_DN | 102 | 6 | 5 | 5.2991 | 0.0026 |
| MIKKELSEN_ES_LCP_WITH_H3K4ME3 | 59 | 5 | 3 | 3.5425 | 0.0026 |
| GHANDHI_BYSTANDER_IRRADIATION_UP | 73 | 4 | 4 | 4.9514 | 0.0025 |
| ALTEMEIER_RESPONSE_TO_LPS_WITH_MECHANICAL_VENTILATION | 127 | 7 | 6 | 5.6394 | 0.0025 |
| GALINDO_IMMUNE_RESPONSE_TO_ENTEROTOXIN | 85 | 4 | 5 | 5.2991 | 0.0025 |
| POTTI_TOPOTECAN_SENSITIVITY | 119 | 5 | 7 | 5.9313 | 0.0025 |
| ZAMORA_NOS2_TARGETS_DN | 97 | 4 | 6 | 5.6394 | 0.0024 |
| PID_CXCR4_PATHWAY | 109 | 5 | 6 | 5.6394 | 0.0024 |
| KEEN_RESPONSE_TO_ROSIGLITAZONE_DN | 109 | 5 | 6 | 5.6394 | 0.0024 |
| GAURNIER_PSMD4_TARGETS | 73 | 7 | 3 | 3.5715 | 0.0024 |
| NAKAYAMA_SOFT_TISSUE_TUMORS_PCA1_UP | 70 | 7 | 3 | 3.2666 | 0.0024 |
| BROWNE_HCMV_INFECTION_8HR_UP | 99 | 4 | 6 | 5.6394 | 0.0023 |
| REACTOME_L1CAM_INTERACTIONS | 87 | 4 | 5 | 5.0232 | 0.0022 |
| KEGG_CELL_ADHESION_MOLECULES_CAMS | 135 | 9 | 5 | 5.0232 | 0.0021 |
| KEGG_TOLL_LIKE_RECEPTOR_SIGNALING_PATHWAY | 108 | 5 | 5 | 5.2991 | 0.0019 |
| BASSO_CD40_SIGNALING_UP | 97 | 4 | 5 | 5.2991 | 0.0019 |
| GHANDHI_DIRECT_IRRADIATION_UP | 97 | 4 | 5 | 5.2991 | 0.0019 |
| REACTOME_PLATELET_ACTIVATION_SIGNALING_AND_AGGREGATION | 201 | 13 | 6 | 5.6394 | 0.0018 |
| REACTOME_HEMOSTASIS | 251 | 16 | 7 | 5.9313 | 0.0018 |
| SANA_TNF_SIGNALING_UP | 79 | 5 | 3 | 4.3352 | 0.0018 |
| WOOD_EBV_EBNA1_TARGETS_UP | 114 | 4 | 6 | 5.5910 | 0.0017 |
| MIKKELSEN_IPS_LCP_WITH_H3K4ME3 | 98 | 5 | 4 | 4.9514 | 0.0017 |
| MCLACHLAN_DENTAL_CARIES_DN | 230 | 13 | 7 | 5.9313 | 0.0017 |
| PROVENZANI_METASTASIS_DN | 130 | 4 | 7 | 5.9313 | 0.0017 |
| MCLACHLAN_DENTAL_CARIES_UP | 236 | 13 | 7 | 5.9313 | 0.0016 |
| MISSIAGLIA_REGULATED_BY_METHYLATION_UP | 121 | 5 | 5 | 5.2991 | 0.0015 |
| LANDIS_ERBB2_BREAST_TUMORS_324_DN | 141 | 4 | 7 | 5.9313 | 0.0014 |
| VART_KSHV_INFECTION_ANGIOGENIC_MARKERS_DN | 141 | 4 | 7 | 5.9313 | 0.0014 |
| BOQUEST_STEM_CELL_DN | 191 | 9 | 6 | 5.6394 | 0.0014 |
| VART_KSHV_INFECTION_ANGIOGENIC_MARKERS_UP | 168 | 7 | 6 | 5.5910 | 0.0014 |
| ICHIBA_GRAFT_VERSUS_HOST_DISEASE_35D_UP | 128 | 6 | 5 | 4.5281 | 0.0014 |
| WIERENGA_STAT5A_TARGETS_DN | 157 | 6 | 6 | 5.6394 | 0.0014 |
| KEGG_DILATED_CARDIOMYOPATHY | 94 | 6 | 3 | 4.0375 | 0.0014 |
| LENAOUR_DENDRITIC_CELL_MATURATION_DN | 127 | 5 | 5 | 5.2991 | 0.0014 |
| IWANAGA_CARCINOGENESIS_BY_KRAS_PTEN_UP | 145 | 4 | 7 | 5.9313 | 0.0013 |
| KOKKINAKIS_METHIONINE_DEPRIVATION_48HR_UP | 135 | 4 | 6 | 5.5910 | 0.0012 |
| ROME_INSULIN_TARGETS_IN_MUSCLE_DN | 114 | 4 | 5 | 4.6698 | 0.0012 |
| KEGG_REGULATION_OF_ACTIN_CYTOSKELETON | 218 | 8 | 7 | 5.9313 | 0.0012 |
| THUM_SYSTOLIC_HEART_FAILURE_UP | 219 | 8 | 7 | 5.9313 | 0.0012 |
| ZHOU_INFLAMMATORY_RESPONSE_FIMA_UP | 176 | 5 | 7 | 5.9313 | 0.0011 |
| LINSLEY_MIR16_TARGETS | 158 | 4 | 7 | 5.9313 | 0.0011 |
| KEGG_SYSTEMIC_LUPUS_ERYTHEMATOSUS | 127 | 4 | 5 | 5.2918 | 0.0011 |
| ACEVEDO_METHYLATED_IN_LIVER_CANCER_DN | 160 | 4 | 7 | 5.9313 | 0.0011 |
| JISON_SICKLE_CELL_DISEASE_UP | 180 | 5 | 7 | 5.9313 | 0.0011 |
| KIM_WT1_TARGETS_12HR_UP | 147 | 4 | 6 | 5.5910 | 0.0010 |
| VERHAAK_AML_WITH_NPM1_MUTATED_UP | 173 | 7 | 5 | 5.2918 | 0.0010 |
| OSWALD_HEMATOPOIETIC_STEM_CELL_IN_COLLAGEN_GEL_UP | 219 | 7 | 7 | 5.9313 | 0.0010 |
| WALLACE_PROSTATE_CANCER_RACE_UP | 219 | 7 | 7 | 5.9313 | 0.0010 |
| PETROVA_ENDOTHELIUM_LYMPHATIC_VS_BLOOD_DN | 163 | 6 | 5 | 5.2991 | 0.0010 |
| BOCHKIS_FOXA2_TARGETS | 206 | 6 | 7 | 5.9313 | 0.0010 |
| PILON_KLF1_TARGETS_UP | 207 | 6 | 7 | 5.9313 | 0.0010 |
| HSIAO_LIVER_SPECIFIC_GENES | 243 | 10 | 6 | 5.5837 | 0.0010 |
| CONCANNON_APOPTOSIS_BY_EPOXOMICIN_UP | 211 | 6 | 7 | 5.9313 | 0.0009 |
| CAIRO_LIVER_DEVELOPMENT_DN | 208 | 7 | 6 | 5.6394 | 0.0009 |
| FOSTER_KDM1A_TARGETS_UP | 176 | 4 | 7 | 5.9313 | 0.0009 |
| HAN_SATB1_TARGETS_UP | 220 | 6 | 7 | 5.9313 | 0.0009 |
| YANG_BCL3_TARGETS_UP | 222 | 6 | 7 | 5.9313 | 0.0009 |
| WINTER_HYPOXIA_METAGENE | 245 | 7 | 7 | 5.9313 | 0.0008 |
| ZWANG_CLASS_3_TRANSIENTLY_INDUCED_BY_EGF | 187 | 5 | 6 | 5.6394 | 0.0008 |
| KEGG_FOCAL_ADHESION | 205 | 6 | 6 | 5.6394 | 0.0008 |
| HELLER_HDAC_TARGETS_DN | 229 | 6 | 7 | 5.9313 | 0.0008 |
| BENPORATH_MYC_TARGETS_WITH_EBOX | 230 | 6 | 7 | 5.9313 | 0.0008 |
| SWEET_LUNG_CANCER_KRAS_UP | 234 | 6 | 7 | 5.9313 | 0.0008 |
| WANG_CISPLATIN_RESPONSE_AND_XPC_UP | 193 | 4 | 7 | 5.9313 | 0.0008 |
| MCBRYAN_PUBERTAL_BREAST_4_5WK_UP | 231 | 7 | 6 | 5.6394 | 0.0007 |
| SHEDDEN_LUNG_CANCER_GOOD_SURVIVAL_A4 | 154 | 4 | 5 | 5.2434 | 0.0007 |
| GRAESSMANN_RESPONSE_TO_MC_AND_DOXORUBICIN_UP | 198 | 4 | 7 | 5.9313 | 0.0007 |
| RIGGI_EWING_SARCOMA_PROGENITOR_UP | 199 | 4 | 7 | 5.9313 | 0.0007 |
| RODWELL_AGING_KIDNEY_UP | 195 | 6 | 5 | 5.2991 | 0.0007 |
| KAYO_AGING_MUSCLE_UP | 223 | 5 | 7 | 5.9313 | 0.0007 |
| FER0DEZ_BOUND_BY_MYC | 181 | 4 | 6 | 5.6394 | 0.0007 |
| YOSHIMURA_MAPK8_TARGETS_UP | 228 | 5 | 7 | 5.9313 | 0.0007 |
| KEGG_ENDOCYTOSIS | 179 | 4 | 6 | 5.3151 | 0.0007 |
| HAMAI_APOPTOSIS_VIA_TRAIL_DN | 142 | 4 | 4 | 4.9514 | 0.0007 |
| CHEN_METABOLIC_SYNDROM_NETWORK | 207 | 4 | 7 | 5.9313 | 0.0007 |
| BROWNE_HCMV_INFECTION_14HR_DN | 232 | 5 | 7 | 5.9313 | 0.0007 |
| HIRSCH_CELLULAR_TRANSFORMATION_SIGNATURE_UP | 230 | 6 | 6 | 5.5910 | 0.0006 |
| HELLER_SILENCED_BY_METHYLATION_UP | 231 | 6 | 6 | 5.6394 | 0.0006 |
| ENK_UV_RESPONSE_EPIDERMIS_UP | 236 | 5 | 7 | 5.9313 | 0.0006 |
| TONKS_TARGETS_OF_RUNX1_RUNX1T1_FUSION_HSC_DN | 172 | 4 | 5 | 5.2991 | 0.0006 |
| PICCALUGA_ANGIOIMMUNOBLASTIC_LYMPHOMA_UP | 195 | 4 | 6 | 5.6394 | 0.0006 |
| BENPORATH_ES_WITH_H3K27ME3 | 195 | 4 | 6 | 5.6394 | 0.0006 |
| SATO_SILENCED_BY_METHYLATION_IN_PANCREATIC_CANCER_1 | 218 | 4 | 7 | 5.9313 | 0.0006 |
| PLASARI_TGFB1_TARGETS_10HR_UP | 175 | 4 | 5 | 5.2991 | 0.0006 |
| VERHAAK_GLIOBLASTOMA_NEURAL | 198 | 4 | 6 | 5.6394 | 0.0006 |
| PUJANA_ATM_PCC_NETWORK | 222 | 4 | 7 | 5.9313 | 0.0006 |
| DELYS_THYROID_CANCER_UP | 247 | 6 | 6 | 5.5910 | 0.0006 |
| HELLER_HDAC_TARGETS_SILENCED_BY_METHYLATION_DN | 227 | 5 | 6 | 5.5910 | 0.0005 |
| REACTOME_CYTOKINE_SIGNALING_IN_IMMUNE_SYSTEM | 253 | 5 | 7 | 5.9313 | 0.0005 |
| LINDGREN_BLADDER_CANCER_CLUSTER_2B | 230 | 5 | 6 | 5.5910 | 0.0005 |
| SMID_BREAST_CANCER_NORMAL_LIKE_UP | 232 | 5 | 6 | 5.6394 | 0.0005 |
| LEI_MYB_TARGETS | 258 | 5 | 7 | 5.9313 | 0.0005 |
| BUYTAERT_PHOTODYNAMIC_THERAPY_STRESS_DN | 208 | 4 | 6 | 5.5910 | 0.0005 |
| ACEVEDO_LIVER_TUMOR_VS_NORMAL_ADJACENT_TISSUE_DN | 208 | 4 | 6 | 5.5910 | 0.0005 |
| GAL_LEUKEMIC_STEM_CELL_DN | 209 | 4 | 6 | 5.6394 | 0.0005 |
| JAATINEN_HEMATOPOIETIC_STEM_CELL_DN | 209 | 4 | 6 | 5.6394 | 0.0005 |
| NUYTTEN_EZH2_TARGETS_UP | 208 | 4 | 6 | 5.5837 | 0.0005 |
| RUTELLA_RESPONSE_TO_HGF_UP | 233 | 5 | 6 | 5.5910 | 0.0005 |
| TARTE_PLASMA_CELL_VS_PLASMABLAST_UP | 232 | 4 | 7 | 5.9313 | 0.0005 |
| MARTORIATI_MDM4_TARGETS_FETAL_LIVER_DN | 235 | 4 | 7 | 5.9313 | 0.0005 |
| HOSHIDA_LIVER_CANCER_SUBCLASS_S1 | 235 | 4 | 7 | 5.9313 | 0.0005 |
| SANSOM_APC_TARGETS_DN | 236 | 4 | 7 | 5.9313 | 0.0005 |
| COLINA_TARGETS_OF_4EBP1_AND_4EBP2 | 242 | 4 | 7 | 5.9313 | 0.0005 |
| ENK_UV_RESPONSE_EPIDERMIS_DN | 244 | 4 | 7 | 5.9313 | 0.0005 |
| YOSHIMURA_MAPK8_TARGETS_DN | 245 | 4 | 7 | 5.9313 | 0.0005 |
| REACTOME_ADAPTIVE_IMMUNE_SYSTEM | 249 | 4 | 7 | 5.9313 | 0.0005 |
| PHONG_TNF_RESPONSE_NOT_VIA_P38 | 227 | 4 | 6 | 5.6394 | 0.0004 |
| CAIRO_HEPATOBLASTOMA_DN | 227 | 4 | 6 | 5.5910 | 0.0004 |
| HOSHIDA_LIVER_CANCER_SUBCLASS_S3 | 255 | 5 | 6 | 5.6394 | 0.0004 |
| REACTOME_DEVELOPMENTAL_BIOLOGY | 258 | 4 | 7 | 5.9313 | 0.0004 |
| ALFANO_MYC_TARGETS | 237 | 4 | 6 | 5.6394 | 0.0004 |
| REACTOME_AXON_GUIDANCE | 245 | 4 | 6 | 5.6394 | 0.0004 |
| QI_PLASMACYTOMA_UP | 251 | 4 | 6 | 5.6394 | 0.0004 |
| KEGG_PATHWAYS_IN_CANCER | 259 | 4 | 6 | 5.6394 | 0.0003 |
| KEGG_MAPK_SIGNALING_PATHWAY | 261 | 5 | 5 | 5.2991 | 0.0003 |
| SCHUETZ_BREAST_CANCER_DUCTAL_INVASIVE_UP | 240 | 4 | 5 | 5.2991 | 0.0003 |
